# Network representation of multicellular activity in pancreatic islets: Technical considerations for functional connectivity analysis

**DOI:** 10.1371/journal.pcbi.1012130

**Published:** 2024-05-13

**Authors:** Marko Šterk, Yaowen Zhang, Viljem Pohorec, Eva Paradiž Leitgeb, Jurij Dolenšek, Richard K. P. Benninger, Andraž Stožer, Vira Kravets, Marko Gosak

**Affiliations:** 1 Faculty of Natural Sciences and Mathematics, University of Maribor, Maribor, Slovenia; 2 Faculty of Medicine, University of Maribor, Maribor, Slovenia; 3 Department of Pediatrics, Department of Bioengineering, University of California, San Diego, La Jolla, California, United States of America; 4 Department of Bioengineering, Barbara Davis Center for Diabetes, Aurora, Colorado, United States of America; 5 Barbara Davis Center for Childhood Diabetes, University of Colorado Anschutz Medical Campus, Aurora, Colorado, United States of America; 6 Department of Bioengineering, Jacobs School of Engineering, University of California, San Diego, La Jolla, California, United States of America; 7 Alma Mater Europaea, Maribor; University of Pittsburgh, UNITED STATES

## Abstract

Within the islets of Langerhans, beta cells orchestrate synchronized insulin secretion, a pivotal aspect of metabolic homeostasis. Despite the inherent heterogeneity and multimodal activity of individual cells, intercellular coupling acts as a homogenizing force, enabling coordinated responses through the propagation of intercellular waves. Disruptions in this coordination are implicated in irregular insulin secretion, a hallmark of diabetes. Recently, innovative approaches, such as integrating multicellular calcium imaging with network analysis, have emerged for a quantitative assessment of the cellular activity in islets. However, different groups use distinct experimental preparations, microscopic techniques, apply different methods to process the measured signals and use various methods to derive functional connectivity patterns. This makes comparisons between findings and their integration into a bigger picture difficult and has led to disputes in functional connectivity interpretations. To address these issues, we present here a systematic analysis of how different approaches influence the network representation of islet activity. Our findings show that the choice of methods used to construct networks is not crucial, although care is needed when combining data from different islets. Conversely, the conclusions drawn from network analysis can be heavily affected by the pre-processing of the time series, the type of the oscillatory component in the signals, and by the experimental preparation. Our tutorial-like investigation aims to resolve interpretational issues, reconcile conflicting views, advance functional implications, and encourage researchers to adopt connectivity analysis. As we conclude, we outline challenges for future research, emphasizing the broader applicability of our conclusions to other tissues exhibiting complex multicellular dynamics.

## Author summary

Islets of Langerhans, multicellular microorgans in the pancreas, are pivotal for whole-body energy homeostasis. Hundreds of beta cells within these networks synchronize to produce insulin, a crucial hormone for metabolic control. Coordinated activity disruptions in these multicellular networks contribute to irregular insulin secretion, a hallmark of diabetes. Recognizing the significance of collective activity, network science approaches have been increasingly applied in islet research. However, variations in experimental setups, imaging techniques, signal processing, and connectivity analysis methods across different research groups pose challenges for integrating findings into a comprehensive picture. Therefore, we present here a systematic analysis of various approaches impacting results in islet activity network representation. We find that methods for constructing functional connectivity maps aren’t critical, but caution is necessary when aggregating data from different islets. Network analysis conclusions are notably influenced by factors such as time series pre-processing, the oscillatory component of signals, and experimental preparation. Despite these challenges, this paper advocates for the adoption of connectivity analysis in future islet research, emphasizing that the insights gained extend beyond pancreatic islets to provide valuable contributions for understanding connectivity in other multicellular systems.

## Introduction

Proper insulin secretion and insulin sensitivity of peripheral tissues are crucial in regulating uptake and disposal of energy rich molecules, thereby sustaining metabolic homeostasis [1]. Pancreatic beta cells constitute part of a crucial negative feedback loop, sensing changes in plasma levels of energy-rich nutrients and accordingly adjusting release of insulin into the bloodstream [2]. The cascade of cellular events connecting the changes in plasma nutrient levels with the proper insulin secretion have been studied in detail [3–10]. The crucial steps in the stimulus-secretion coupling cascade involve an increase in intracellular ATP concentration, closure of ATP-sensitive potassium channels, membrane depolarization, opening of voltage-activated Ca^2+^ channels and an increase in intracellular calcium concentration ([Ca^2+^]_i_), leading ultimately to exocytosis of insulin-containing vesicles. Beta cell response is further modulated by homo- and heterologous cell-to-cell interactions within islets [11–14], by autonomous nerve control [15–17], and by hormones released by the gut [18–20]. Of importance, beta cells display complex oscillatory activity and are intrinsically heterogeneous [8,21,22], with differences observed on molecular [23], morphological [24,25] and functional levels [26,27], and it is only due to strong coupling within the islets that beta cells properly respond to glucose excursions.

In a coupled system of beta cells, glucose stimulation triggers two distinct and qualitatively different phases [8,27–32]. The initial response consists of a phasic increase in activity, characterized by membrane depolarization and increase in [Ca^2+^]_i_ which occurs sooner in higher glucose concentrations [27,33]. In case that the stimulus is still present, a complex tonic activity follows. This second phase is characterized by repetitive membrane potential and [Ca^2+^]_i_ oscillations, as well as pulses of insulin secretion. These oscillations are not generated randomly among cells. Rather, they are phase-lagged between cells, such that waves of membrane depolarization and [Ca^2+^]_i_ are formed, spreading from cell to cell from different wave-initiating cells near the islet periphery [13,16,34–36]. An increase in glucose concentration is coded as a fractional increase in activity within a time period, termed also relative active time [16,27,33] or duty cycle. The mechanistic substrate for such cohesive functioning of beta cells is intercellular communication via gap junction channels, consisting of connexin 36 (Cx36) [34,37–40]. Cx36 provides both metabolic and electrical coupling between spatially organized heterogenous beta cells [34,39,41]. While other mechanism, such as autonomic innervation [42], autocrine and paracrine [43,44] signaling also contribute to cell-cell communication, Cx36 have been shown to play the main role in synchronizing beta cell collectives and maintaining proper insulin secretion [45,46]. Indeed, expression of Cx36 is decreased in diabetic conditions [47,48] leading to desynchronisation of [Ca^2+^]_i_ oscillations and perturbations in pulsatile insulin secretion [37,40,49–52]. Hence, the gap-junctional connections among beta cells are imperative for optimal beta cell function and comprehending their collective dynamics holds significance in elucidating the mechanisms underlying diabetes pathogenesis and its treatment.

Due to their highly heterogeneous nature, the presence of distinct subpopulations, and an ever-changing environment, beta cells display intricate yet coherent intercellular activity patterns [8,53]. Because coordinated intercellular activity is not only crucial for tightly regulated insulin secretion but is also known to be altered in diabetes, researchers are investing considerable effort in describing and studying how collective rhythmicity is established in beta cell populations and how the underlying mechanisms change in disease. In recent years, the emergence of network analyses has provided a promising tool for evaluating data obtained through advanced multicellular imaging, with the goal to objectively characterize collective activity in islets [16,42,54–58]. In this approach, individual cells serve as nodes, and their positions correspond to their physical locations within the tissue. The connections between cells reflect functional associations and are determined based on the temporal similarity of the measured cellular dynamics, most often [Ca^2+^]_i_ activity [56]. The application of network approaches has uncovered a modular organization in the functional beta cell networks, that exhibit greater heterogeneity than anticipated in a gap junction coupled syncytium. The identified indicators of small-worldness and a heavy-tailed degree distribution imply the existence of highly connected cells, called hubs [54,56]. Although their precise function remains somewhat enigmatic, these hubs are believed to represent a subpopulation with distinct attributes that confer upon them an above-average impact on the synchronized behavior [8,13,55,59–61]. Furthermore, the collective responses to stimulation and the mediation of intercellular signals were also found to be influenced by other beta cell subpopulations. Specifically, the first responder cells were found crucial in mediating the responses to increasing stimulation during first phase of the islet’s response [57], whilst the wave initiator cells act as triggers of intercellular signals that synchronize the cells [13,34,62], being thereby presumably implicated in the regulation of pulsatile insulin release during the second phase [14,41]. In recent years, advanced methodological approaches, including optogenetics, photopharmacological methods, and RNA sequencing, along with network analyses, have unveiled specific characteristics within these subpopulations [8,13,41,54,63]. Acknowledging their unique attributes and significant contribution to shaping overall islet activity, there is a growing interest in their role in diabetes development [45,54,64].For this reason, it becomes even more important to precisely define these subpopulations, and objectively determine them through network analyses.

Nevertheless, due to variations in experimental preparations, microscopic imaging techniques, the nature of recorded signals, the following signal processing techniques, and the methods for deriving functional connectivity patterns that are employed by different research groups, comparing findings and integrating them into a comprehensive bigger picture becomes challenging even for experts in islet research. Additionally, the introduction of new terminology has further contributed to disputes in data interpretation, as well as to apparent contradictions regarding functional connectivity and the role of different beta cell subpopulations, which can be in part attributed to aforementioned methodological discrepancies [8,13,27,53,65–67]. To at least partly address these issues, we present here a systematic analysis of how different experimental designs and computational approaches impact the results obtained from network representations of multicellular islet activity. Specifically, we analyze how the results are affected by different methods used to evaluate coordinated cellular behavior and network construction, different timescales of observed oscillatory calcium activity, different mouse strains used for tissue slice preparation, and the type of experimental preparation (i.e., tissue slices vs. isolated islets). All of the above represents some of the most prevalent genuine variations due to the diverse nature of work, experimental techniques, and the availability of equipment in laboratories worldwide.

## Results

### The role of different methods for the evaluation of time series similarities

**Fig 1:**
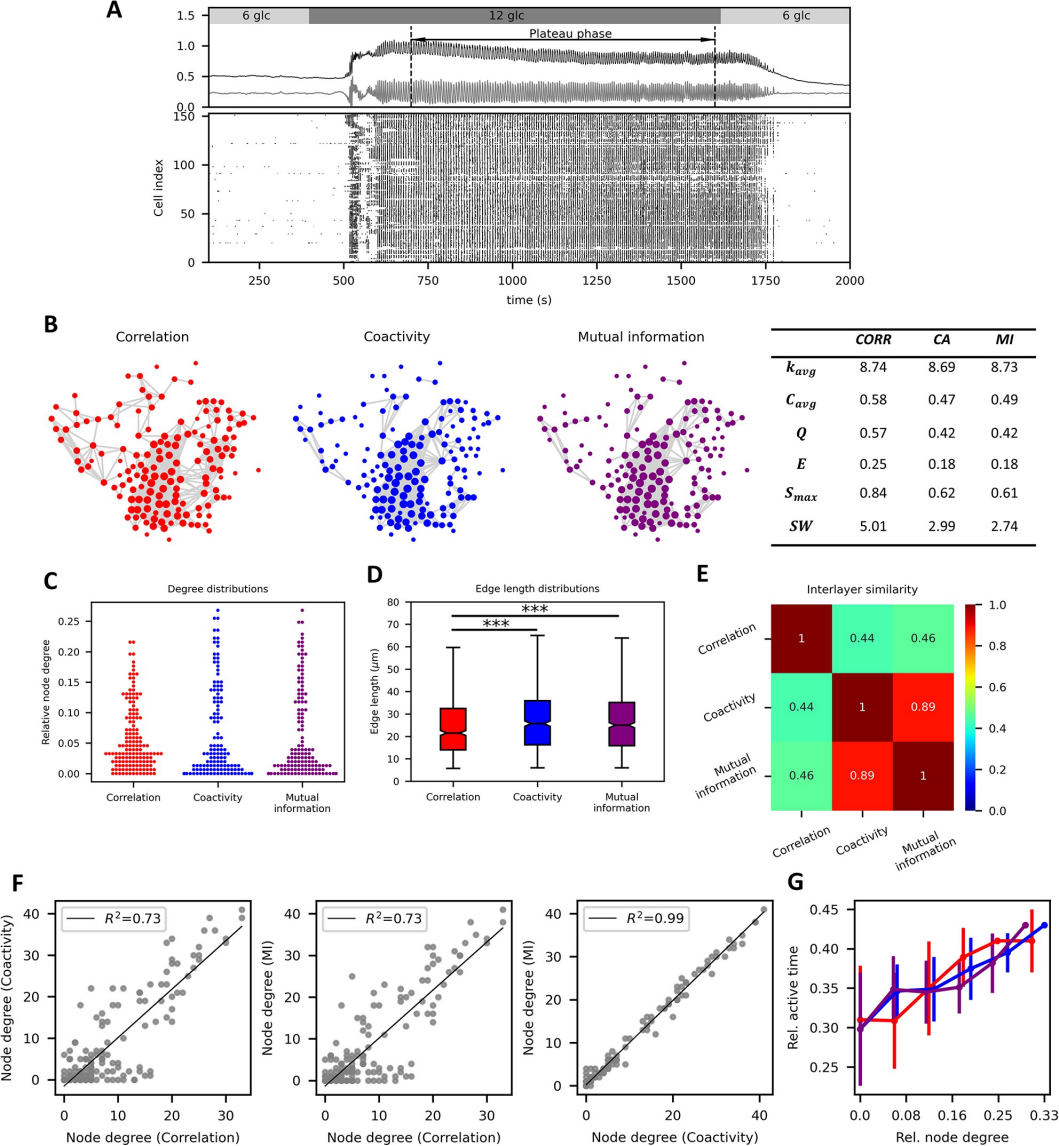
The role of time series similarity measures on the functional beta cell network characteristics. (A) Average signals of unprocessed (black line) and fast oscillatory activity (grey line) (upper panel) and the raster plot (lower panel) showing the binarized fast beta cell dynamics of all cells in the slice. (B) Functional networks designed with fixed average network node degrees (*k*_avg_ ≈ 8) based on three distinct time series similarity measures, and the corresponding network parameters. The correlation method (red) is represented on the left, the coactivity method (blue) in the middle, and the mutual information method (purple) on the right. Colored dots indicate physical locations of cells within islets, while grey lines represent functional connections between them. The table on the right shows the average network node degree (*k*_avg_), average clustering coefficient (*C*_avg_), modularity (*Q*), global efficiency (*E*), relative largest component (*S*_max_), and the small-world coefficient (*SW*) for each network. Degree distributions (C) and distribution of functional connection lengths (D) for networks presented in panel A. Boxes on panels (D) determine the 25^th^ and 75^th^ percentile, whiskers denote the 10^th^ and 90^th^ percentile, and the lines within boxes indicate the median values. E) Jaccard internetwork similarity of the three networks extracted from different methods. (F) Pairwise comparison of node degrees in all networks from panel (B). Gray dots represent the node degree of the same cells in the graphs. (G) Relative active time as a function of node degree in networks built with the correlation method (red), coactivity method (blue) and mutual information method (purple). Dots denote average values and bars denote the standard error.

We start by examining the effect of the type of time series similarity measure used to extract functional beta cell networks. We analyzed the beta cell [Ca^2+^]_i_ responses to glucose stimulation obtained by means of multicellular confocal imaging in acute tissue slices from NMRI mice. The stimulatory glucose concentration was 12 mM and a 15-minute interval of sustained oscillatory activity (i.e., plateau phase) was used for the analysis, as indicated in Fig 1A. Fig 1B shows the extracted functional networks obtained by three different techniques: Pearson correlation coefficient (left panel, red), coactivity coefficient (blue, middle-left panel), and mutual information (purple, middle-right panel). A variable threshold was used so that roughly the same average node degrees (*k*_avg_ between 8 and 9) were obtained in all three networks, facilitating a robust inter-network comparison. The comparison of methods for constructing networks from similarity matrices is analyzed separately in continuation. The right-most panel of Fig 1B shows calculated network parameters. Upon visual inspection of the networks and their corresponding parameters we can see a high degree of similarity between all displayed networks. Consistent with previous findings, all the networks exhibit high levels of clustering, modularity, and small-worldness [16,27,56,68–70]. In Fig 1C and 1D the degree and edge length distributions of the same networks as in Fig 1B are presented, and Fig 1E shows the calculated internetwork similarities (see Methods section for details). All three panels further underline the observed resemblance between the networks extracted from different methods. Furthermore, the similarity in the degree distribution of all three networks suggests comparable variations in the number of functional connections, and, in addition, the level of heterogeneity indicates the presence of hub cells in all three cases.

To investigate the level of similarity between networks further, we present the pair-wise relationships between the node degrees of the same cells in all three constructed networks in Fig 1F. A clear relationship can be observed, with cells having a high degree in one network also having a high degree in the other. There is a consistent trend of matching connection numbers between different networks. The highest overlap is noticed between the coactivity and mutual information, as both these methods rely on binarized signals. Furthermore, previous studies have indicated that there is a tendency that the cells with many functional connections exhibit a higher-than-average activity [13,27]. This characteristic was alluded to when describing hub cell [Ca^2+^]_i_ dynamics as “preceding and outlasting that of the follower cells” in Johnston et al, 2016 [54]. As such, hub cells typically manifest durations of oscillations exceeding the average, which contributes to higher cellular activity. Nevertheless, it is crucial to emphasize that this does not inherently suggest the role of hubs as wave initiators. In other words, the cells with longest oscillations do not necessarily initiate the intercellular waves. Subsequent analyses conducted on more extensive datasets in human [61] and mouse [13] islets revealed a distinct lack of overlap between wave initiators and hubs, although they confirmed that hubs tend to have the longest oscillations. Here, we aimed to confirm whether a comparable relation between relative active time and node degrees can be obtained when employing different techniques to quantify the similarity between [Ca^2+^]_i_ signals. In Fig 1G the relationships between the relative active time and node degree of the same cells are depicted. For all three methods, very similar trends are observed. Specifically, there is a positive relationship between the relative active times of cells and their corresponding node degrees, which indicates that the extracted functional relationships are roughly independent of the methods used to evaluate synchronous cellular activity.

### The role of different techniques used for network construction

**Fig 2:**
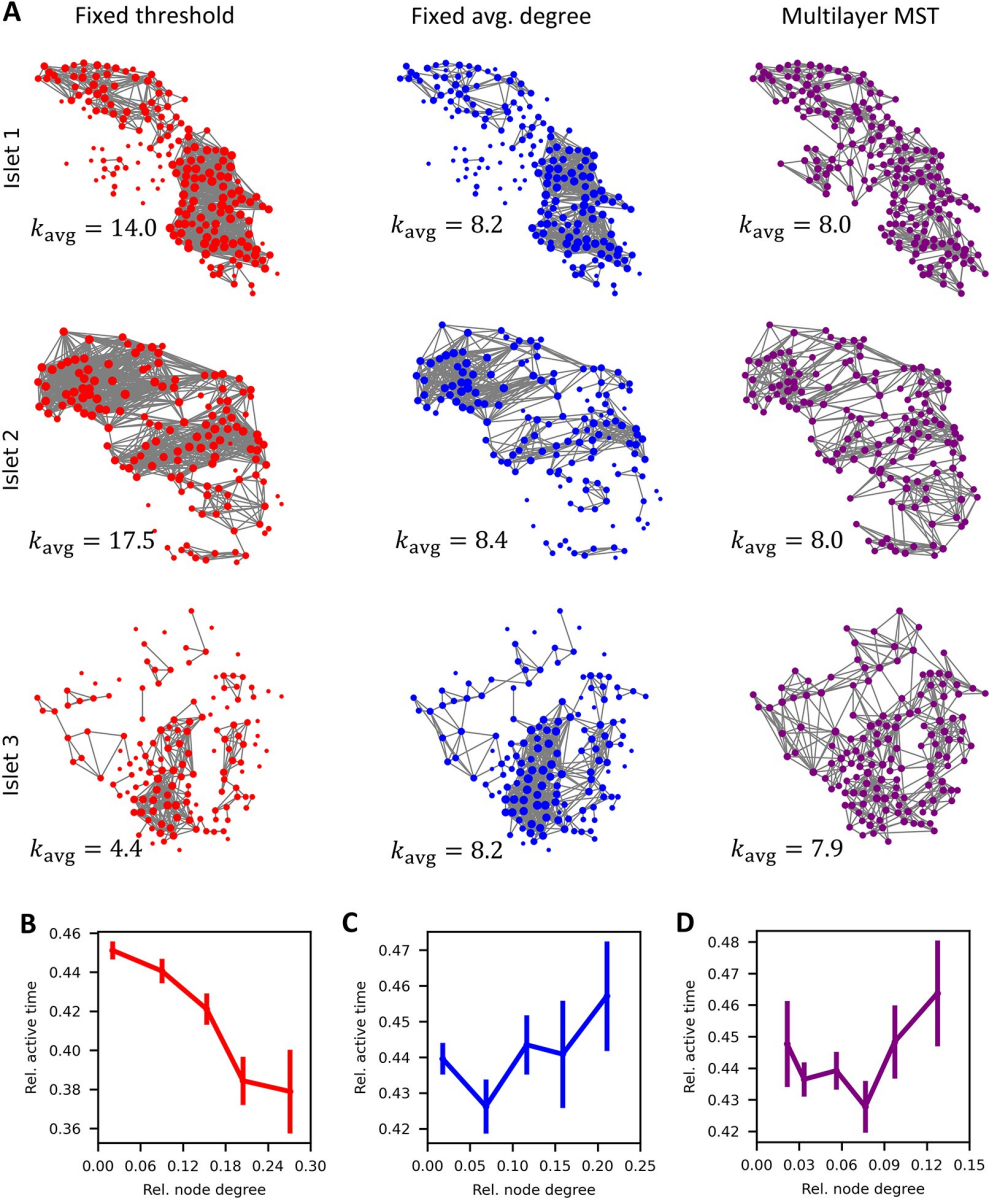
The role of network construction method in determining the beta cell network structure and the relationship to cellular activity parameters. (A) Networks of three different islets (rows) constructed with three distinct construction methods (columns) with indicated average network node degrees (*k*_avg_). Construction methods are fixed correlation threshold (left), fixed average network node degree (middle), and multilayer minimum-spanning tree (right). (B-D) Relative active time of cells as a function of the relative node degree for networks constructed with the fixed correlation threshold method (B; red), fixed average network node degree method (C; blue) and multilayer minimum-spanning tree method (D; purple). Dots indicate the average values of cells within the same degree intervals and the error bars the corresponding SE. Note that the node degrees were normalized to facilitate the comparison of different islets.

Since no significant differences were found among various methods for evaluating intercellular synchronicity, we proceeded with the correlation method in subsequent analyses, which also has the advantage of not requiring signal binarization. In Fig 2 we explore the influence of network construction methods on the functional beta cell network structure and their relationship with the cellular activity parameter. Fig 2A displays functional networks of three different islets (rows) constructed using a fixed similarity threshold (left column), fixed average node degree (middle column), and the multilayer minimum spanning tree (MST) technique (right column). Due to the differences in [Ca^2+^]_i_ signals, such as differences in overall activity, nature of [Ca^2+^]_i_ signals (e.g., fast electrical vs. slow metabolic oscillations, see below for more details), presence of noise, etc., the average node degrees of networks constructed with the fixed similarity thresholds can vary greatly (between 4.4 and 17.5 in the three islets analyzed, see red networks in Fig 2A), whereas the average degrees for the other two techniques are fixed around 8 (see blue and purple networks in Fig 2A). In Fig 2B, 2C and 2D, we present the relationships between the relative active times for the data pooled from all three islets. Notably, a positive correlation between node degree and relative active time is inferred only for the variable threshold, i.e., a fixed average degree, and multilayer MST techniques. This tendency of hub cells exhibiting higher-than-average activity is in accordance with previous reports. In contrast, this relationship is apparently blurred for the fixed threshold technique due to variations in the number of connections along with differences in intrinsic activities among different islets, and hence even an opposite trend can be obtained, despite the fact that the relation is positive in all individual islets.

**Fig 3:**
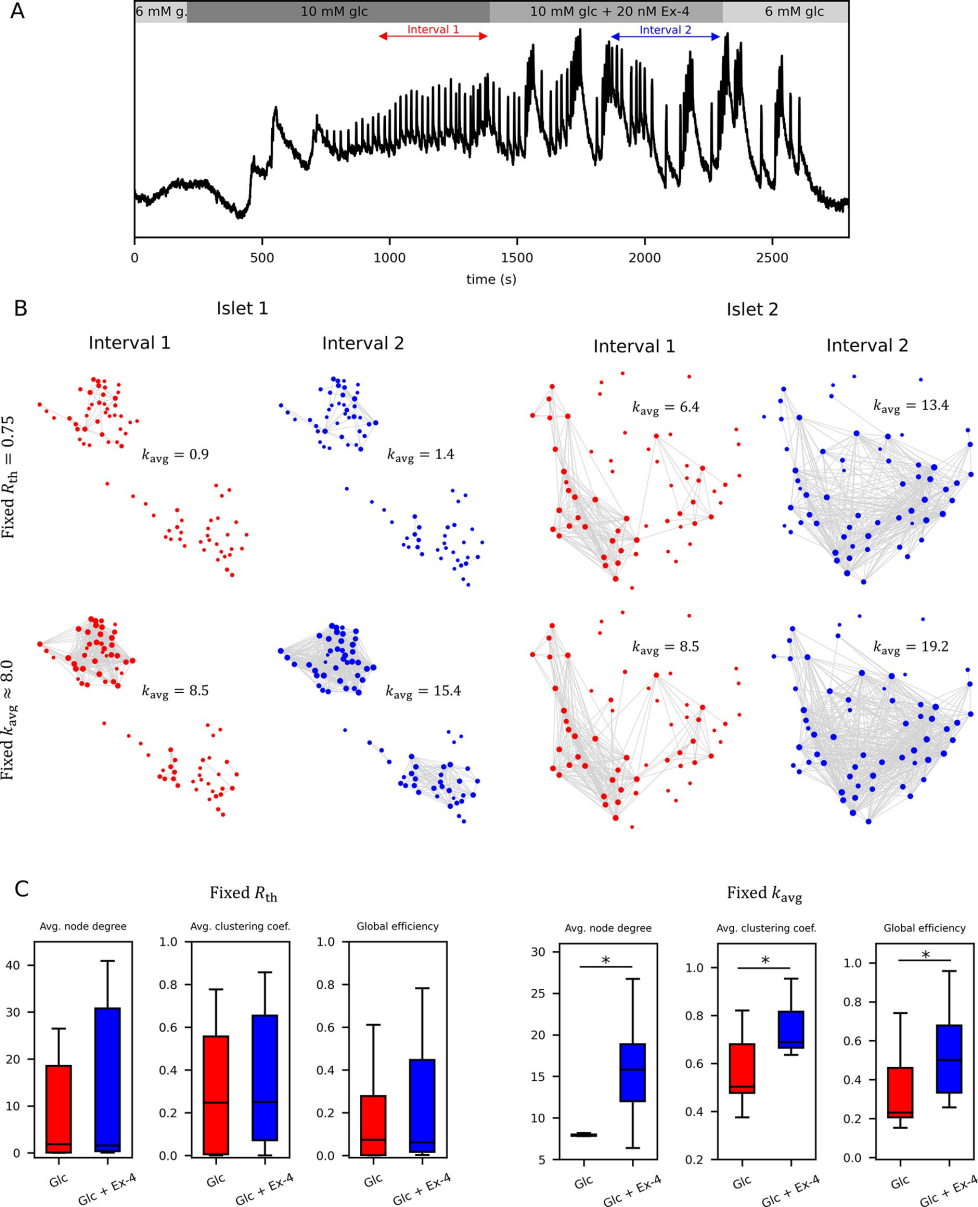
The role if different methods for designing and quantitative analysis of functional beta cell networks. A) Average Ca^2+^ signals from all cells within a representative islet are depicted, stimulated with 10 mM glucose and 10 mM glucose + the GLP-1 receptor agonist exendin-4 (Ex-4), with specified intervals for network analysis. B) Functional networks were constructed for two intervals (Interval 1: 10 mM glucose only; Interval 2: 10 mM glucose + 20 nM Ex-4) across two different islets, utilizing two distinct network design techniques (fixed *R*_th_ = 0.75 and fixed average degree with *k*_avg_ (Int. 1) = 8). The use of the fixed *R*_th_ method resulted in significant variations in network density between the two islets, complicating the comparison of network metrics. Conversely, fixing the average degree in Interval 1 normalized inherent differences in overall coherence of intercellular Ca^2+^ activity, facilitating the assessment of the pharmacological manipulation effect across different islets. C) To illustrate the issue posed by the fixed *R*_th_ method and the resulting high disparities in network densities, we compared the pooling of data from 10 islets subjected to closely matched protocols using both thresholding techniques (i.e., fixed *R*_th_ and fixed *k*_avg_). While both methods revealed a denser network in Interval 2 in response to Ex-4, these differences were almost completely masked by inter-islet variability inherent in the fixed *R*_th_ method. In such scenarios, normalizing the average degree proves to be the superior approach, as it facilitates a robust evaluation of data from different islets. Data used for this analysis is from Ref [71].

To further illustrate the challenge of pooling data from different islets, we present experimental data in Fig 3, where islets were subjected to stimulatory glucose in the first interval and subsequently treated with the GLP-1 receptor agonist exendin-4 (Ex-4) in the second interval. The precise protocol and the average unprocessed Ca^2+^ signal of all cells is shown in Fig 3A. Previous research has demonstrated that Ex-4 increases the density of functional beta cell networks [71], and our results validate this observation under both fixed threshold *R*_th_ and fixed average degree *k*_avg_ methods (Fig 3B). However, the fixed *R*_th_ approach encapsulates substantial heterogeneity among islets, obscuring the effects of Ex-4 stimulation when aggregating data across multiple islets, despite discernible trends at the individual islet level. In such scenarios, employing a fixed *k*_avg_ technique proves to be a more appropriate option for analyzing network differences induced by the pharmacological agent. Specifically, utilizing a variable threshold to maintain a consistent average degree in the initial interval mitigates inter-islet heterogeneity. Subsequently applying the same threshold in the second interval enables an unbiased assessment of the pharmacological intervention, normalized by the network characteristics observed in the first interval of each respective islet. This not only facilitates a robust comparison of network parameters across different islets but also ensures a more accurate statistical evaluation, as demonstrated in Fig 3C.

It is worth noting, however, that in some cases, the fixed *R*_th_ method is the more suitable choice. It is known that with gradually increasing glucose concentration, both activity and intercellular communication levels increase, leading to denser functional networks in these cases [72]. When using fixed *k*_avg_ or multilayer MST methods, which impose a specific number of connections regardless of the nature of activity, such networks do not differ in the number of connections, which is incorrect in these scenarios. Moreover, under conditions of low stimulation, an unjustifiably large number of connections is obtained due to, for example, a low threshold, which does not reflect correlations in dynamics but rather random associations. Such an example is illustrated in S1 Fig.

### The role of the mouse strain used in tissue slice preparation

**Fig 4:**
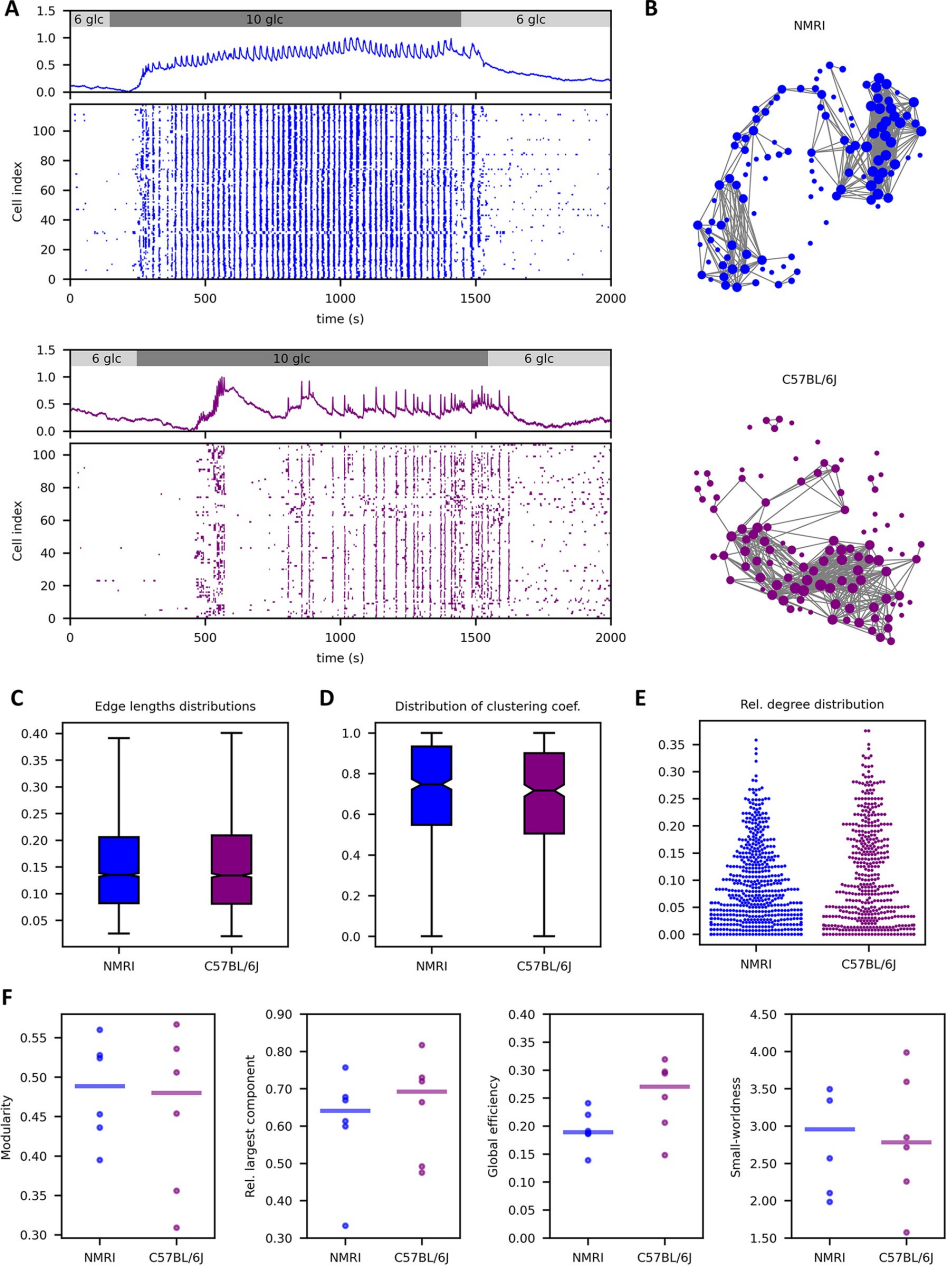
The impact of mouse strain used for tissue slice preparation on the parameters of beta cell networks. (A) Average signals of unprocessed and fast oscillatory activity and the raster plot showing the binarized fast beta cell dynamics of all cells in slices from NMRI mice islets (upper panel, blue) and C57BL/6J mice islets (lower panel, purple). (B) Functional networks derived from representative recordings in islet from NMRI (blue, upper panel) and C57BL/6J (purple, lower panel) mice. (C) Edge length distributions, (D) clustering coefficient distributions, and (E) node degree distributions from a pooled data set from NMRI (blue) and BL6J (purple) mouse recordings. (F) Network parameters for extracted networks from NMRI and BL6J mouse recordings: modularity (left), relative largest component (middle left), global efficiency (middle right), and small-worldness coefficient (right). Dots represent values of individual recordings with horizontal lines indicating median values. Boxes on panels (B) and (C) determine the 25*th* and 75*th* percentile, whiskers denote the 10*th* and 90*th* percentile and the lines within boxes indicate median values. Data was pooled from islets/cells: 6/779 (NMRI), 6/617 (C57BL/6J). In all recordings, the islets were stimulated with 10 mM glucose and 10–20 minute intervals in the plateau phase were used for the analysis.

Laboratory mice are a vital source of islets of Langerhans in beta cell physiology research; however, various laboratories employ various mouse models. Previous research has indicated that there is a considerable phenotypic variation between different mouse strains as well as substrains of the inbred strains [73–75] which manifest themselves also in beta cell responses to glucose and [Ca^2+^]_i_ signalling characteristics [33,76]. For that reason, we investigate here whether the functional beta cell network structure extracted from multicellular [Ca^2+^]_i_ recordings in tissue slices depends on the mouse strain. To this purpose, we compared the beta cell networks from outbred NMRI mice and inbred C57BL/6J mice. We used the correlation method to evaluate similarity between [Ca^2+^]_i_ signals and the fixed average degree method (*k*_avg_ = 8) to construct networks. In all recordings we used a 6-10-6 mM glucose protocol, as presented in Fig 4A. Intervals of 10–20 min sustained activity in the plateau phase were then used for the analysis. In Fig 4B we show typical networks from both strains, which, upon visual inspection, exhibit a rather similar topological organization. To provide a more detailed and quantitative insight, we computed various network metrics from pooled data from multiple islets. Results in Fig 4C, 4D and 4E indicate that the edge length, clustering coefficient, and degree distributions are very similar. Furthermore, the computation of network parameters presented in Fig 4F has revealed that beta cell networks from different mouse strains exhibit a similar degree of functional segregation, efficiency, and small-worldness; none of the results were identified as significant.

### The role of different time scales of oscillatory [Ca^2+^]_i_ activity and time series preparation

**Fig 5:**
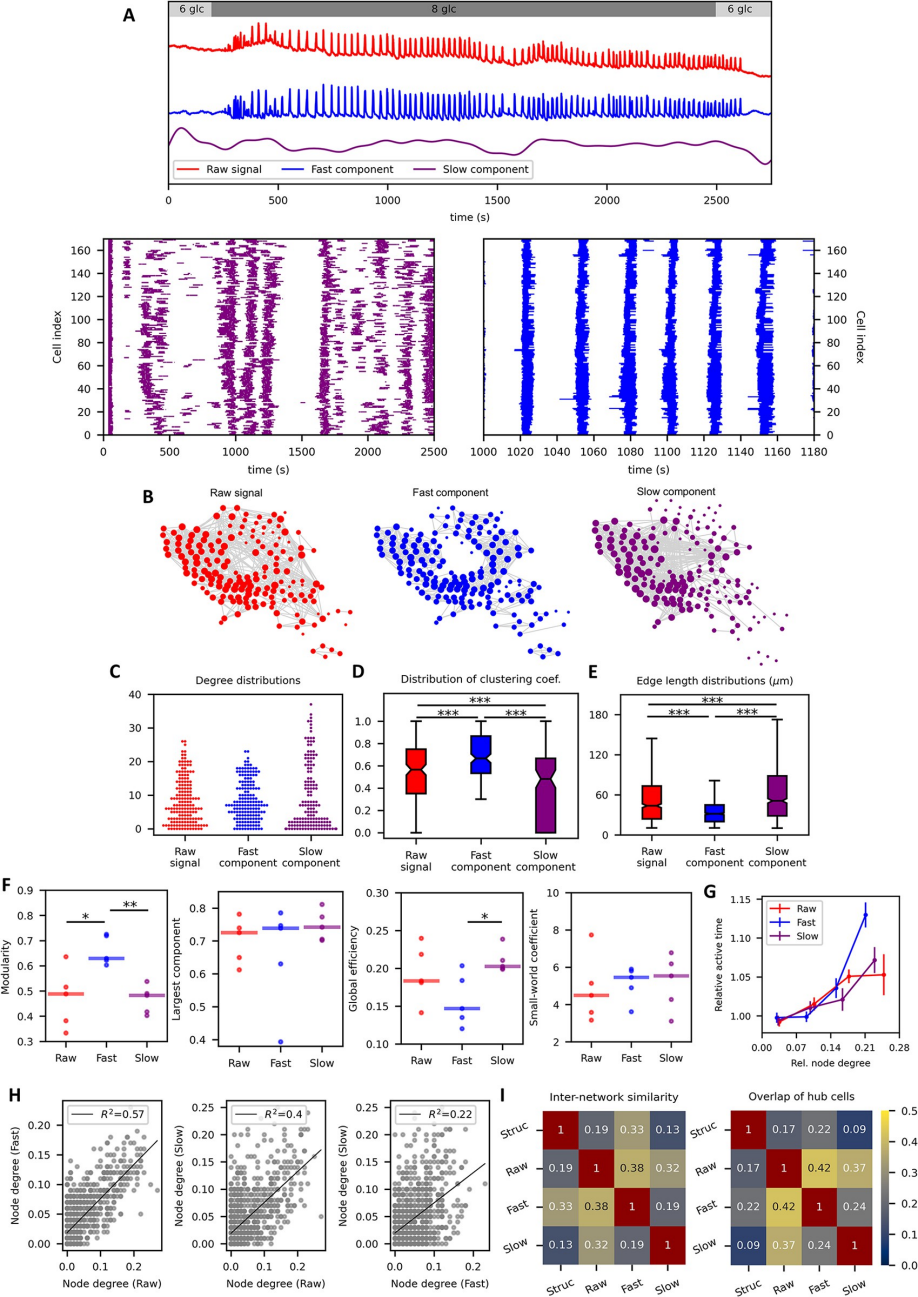
The role of the type of oscillations and signal preparation on the characteristics of functional network topology and the relations to the cellular activity. (A) Unprocessed (red), fast-component only (blue), and slow-component only (purple) average [Ca^2+^]_i_ signal of all cells in the islet from acute tissue slice from NMRI mouse. The lower panels display raster plots that represent the binarized activity of the slow and fast oscillatory components. (B) Functional networks designed based on raw cellular signals (left), fast-component only signals (middle), and slow-component only signals (right). Networks were constructed with the fixed average network node degree method (*k*_*avg*_≈8.0) based on time series correlations as the similarity measure. Distribution of node degrees (C), clustering coefficients (D) and functional connection lengths (E) for the three networks presented in panel B. (F) Network parameters extracted from functional connectivity maps derived from different oscillatory components. (G) Relative active time of cells as a function of their corresponding node degrees in networks constructed based on raw signals (red), fast-component only signals (blue) and slow-component only signals (purple). Colored dots represent average values of cells within the same degree intervals and the error bars denote SE. Individual values were normalized by the average value of the relative active time within the given islet so to ease comparison between different islets. (H) The pairwise relationships between node degrees in different networks. The grey dots denote values from individual cells and the black line indicates the linear fit, whereby *R*^2^ indicates goodness-of-fit. I) Similarity between different types of networks (left) and the relative overlap of hub cells (right), identified as the top 1/6 of the most connected cells. The structural networks were modeled as equivalent geometric networks, in which nearby cells are deemed connected (see Materials and Methods and S4 Fig). Boxes in panels (D) and (E) determine the 25^th^ and 75^th^ percentile, whiskers denote the 10^th^ and 90^th^ percentile and the horizontal lines within boxes indicate the median values. Dots in panel (F) indicate the values from individual islets and the horizontal line denote the median. Stars denote statistical differences; *p<0.05,**p<0.01. Data presented in panels (F-I) is based on 5 different islets.

Next, we investigate how the type of oscillatory activity and signal preparation impact the functional beta cell network topology. To this purpose, we performed prolonged multicellular imaging in tissue slices from NMRI mice. Fig 5A displays an average [Ca^2+^]_i_ signal of all cells in a representative islet under stimulation with 8 mM glucose. Three different temporal traces are presented: the unprocessed (i.e., raw recorded) signal (top, red), the filtered slow oscillations (middle, purple), and the filtered fast oscillations (middle, blue).The fast and slow oscillations principally represent the electrical and metabolic activity of cells, respectively [77]. The lower panels in Fig 5A feature raster plots depicting binarized activity of the slow and fast oscillatory component. Notably, both types of oscillatory activity exhibit distinct, regular patterns. In Fig 5B we present correlation-based functional networks constructed with the fixed average degree technique for the three distinct signal types. A visual assessment points out a clear difference between the three extracted networks. The fast oscillation-based network (middle panel) exhibits shorter edge lengths and a more clustered, localized, structure, while the slow oscillation-based network (right panel) shows more long-range edges and a less clustered structure. A quantitative assessment of the networks confirms the observed differences. The slow oscillatory component network is more heterogeneous, less clustered and exhibits longer functional connections (Fig 5C, 5D and 5E). The reason for this is in the type of cellular dynamics the networks encode. The fast oscillations are representative of the electrical activity of cells, which is mediated by gap-junction-driven intercellular waves and thus contributes to the shorter, more clustered network structure which is quite similar to the underlying physical network. On the other hand, the slow component signal is associated with cellular metabolism which is to a greater extent affected by the similarity of intrinsic metabolic characteristics of cells and less by cell-to-cell coupling [56,70,78,79]. Interestingly, the raw-signal-derived functional network appears to be poised in between, which is somehow expected, as it encompasses both types of oscillatory activity. To evaluate the properties of different networks further, we quantified the extracted functional connectivity patterns using conventional network metrics (Fig 5F). The results indicate that the networks derived from different dynamical components have comparable values of the small-world coefficient and the relative largest component, but there are profound differences in modularity and global efficiency. Namely, the fast oscillations-derived network is more segregated and exhibits lower efficiency, primarily due to the less pronounced long-range connections. Moreover, we present in Fig 5G the relationship between the relative active time of cells and their corresponding node degrees in all three types of networks. The tendency of hub cells being the most active is most pronounced in the case of fast oscillations, whereas the relation is less apparent for the raw and slow component. Notably, the latter aligns with recent theoretical predictions [78]. Finally, we assess the similarities between the three networks and present in Fig 5H the pair-wise relationships between the node degrees in different networks. The results indicate that the strongest relation exists between the fast and raw oscillatory signals, while the relationship is the weakest between the fast and the slow component. To investigate this in further detail, we quantified the overlap between different networks, including the hypothesized structural network that was modeled as a geometric network in which nearby cells are connected. From Fig 5I, we can observe a substantial similarity in both inter-network similarity and overlap of hub cells between the unprocessed signal and the signals of both oscillatory components, with a higher level of similarity observed in the fast component. This is expected in signals from slices, as the fast component is very pronounced. However, the key point is that the highest level of similarity between the structural network and the functional networks is obtained from fast oscillations, while the similarity between the structural and slow networks is substantially lower. Similarly, the connection between the fast and slow component-derived networks is relatively low, as previously indicated by the results in Fig 5H. These quantitative results can be further visually assessed with the illustrations in S2 Fig, depicting all four types of networks for all 5 islets included in the analysis. It can be observed that the networks of unprocessed signals and signals of the slow component contain many long-range connections, while those in the fast component network are significantly fewer, making it visually more similar to the structural network. Importantly, fast oscillations may be more strongly determined by slow oscillations, such as in the case of compound oscillations [80,81]. Such an example is depicted in S3 Fig and in this case, the functional network based on slow oscillations rather than fast oscillations is most similar to the functional network based on the raw signal. However, the functional network based on fast oscillations remains the one that is most similar to the structural network (S3B and S3C Fig).

### Functional connectivity networks in isolated islets

**Fig 6:**
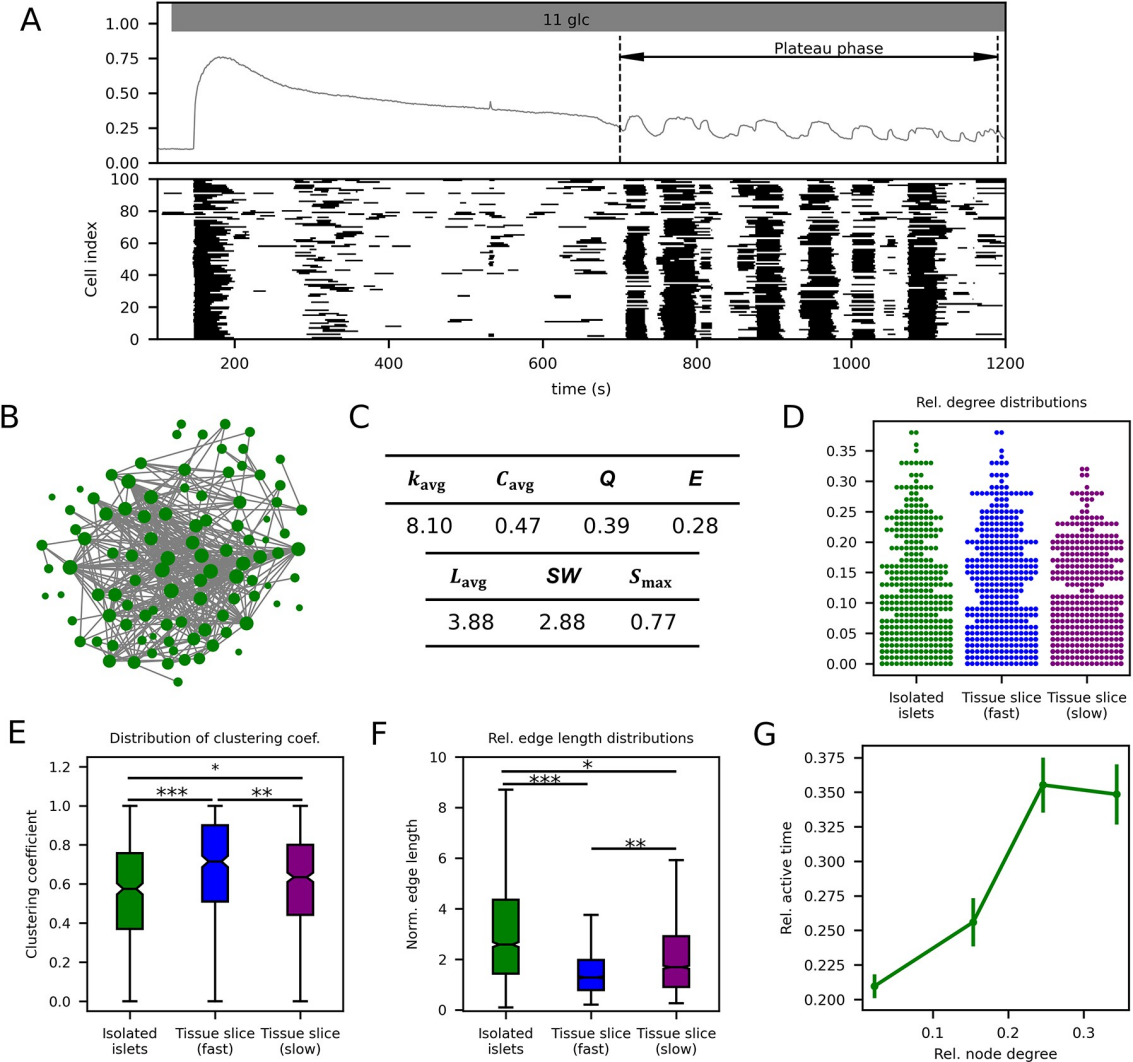
Functional beta cell connectivity patterns in isolated islets. (A) Average [Ca^2+^]_i_ signal of a representative isolated islet recording with indicated plateau phase for signal analysis (upper panel) and corresponding binarized oscillatory activity of all cells in the recording (lower panel). (B) Extracted functional network based on cellular signals in panel (A) constructed with the fixed average network node degree method with an average network node degree *k*_*avg*_≈8.0. Green dots represent physical locations of cells within the islet and grey lines indicate functional connections between them. (C) Extracted average functional network parameters: average network node degree (*k*_*avg*_), average clustering coefficient (*C*_*avg*_), modularity (*Q*), global efficiency (*E*), average shortest path length (*L*_*avg*_), small-world coefficient (*SW*), and relative largest component (*S*_*max*_). Degree distributions of all extracted functional networks (D), and corresponding distributions of clustering coefficients (E), and relative edge lengths (F). To ease comparison between different islets, the physical lengths of connections were normalized with the average distance to the eight nearest neighbors. Additionally, in panels (D-F) data illustrating network attributes derived from fast and slow activities in slices from C57BL/6J mice are presented for comparison. (G) Relative active time as a function of node degree for all extracted functional networks. Boxes on panels (E-F) determine the 25^th^ and 75^th^ percentile, whiskers denote the 10^th^ and 90^th^ percentile, and the lines within boxes indicate the median values. Dots in panel (G) represent average values and vertical bars denote the standard error. Data for panels (C-G) for isolated islets was pooled from islets/cells: 5/468 and for slices the same dataset was used as in Fig 4 (islets/cells: 6/617). *p<0.05,**p<0.01, ***p<0.001.

In addition to acute tissue slices, isolated islets play a prevalent role in beta cell research, including in the context of collective activity network analyses. Thus, we proceed with analyzing the nature of multicellular dynamics and the underlying functional networks within islets isolated from C57BL/6J mice. In Fig 6A, we present the responses of a representative isolated islet upon transitioning from 2 mM to 11 mM glucose. The cells exhibit an initial, profound elevation in [Ca^2+^]_i_ levels, followed by the emergence of coordinated [Ca^2+^]_i_ oscillations after approximately 8–10 minutes. The raster plots indicate that these oscillations frequently span the entire islet. The functional network extracted from the phase of sustained oscillatory activity, constructed based on time series correlation as the similarity measure along with the fixed average network node degree method, is shown in Fig 6B. The characterization of beta cell networks was based on 5 different isolated islets subjected to the same protocol. In the table shown in Fig 6C the average values of network parameters are provided and Fig 6D shows the pooled degree distributions. We can observe that the topological parameters of networks from isolated islets do not differ much from those in slice-based networks: they are quite modular and exhibit features of small-world networks. However, upon visually evaluating the network illustrated in Fig 6B and considering the characteristics of clustering coefficient (Fig 6E) and functional connection length distributions (Fig 6F), it becomes evident that the networks observed in isolated islets exhibit properties that are more similar to the networks characterized by slow activity in slices. Note that for comparison the data on fast and slow activity-derived networks from slices from C57BL/6J mice are provided separately. For this comparison, the same dataset was used as in Fig 4, where also the stimulatory glucose concertation was similar (i.e., 10 mM). In contrast to fast oscillation-based networks in slices, isolated islet networks manifest a higher efficiency, a reduced modularity, and low clustering coefficient values. Moreover, the distribution of relative connection lengths indicates that there is a larger fraction of long-range connections in isolated islets. All these attributes can be observed in slow oscillation-based networks in slices. Notably, within isolated islets, a discernible trend emerges where cells with an increased number of functional connections consistently demonstrate higher relative active times—reminiscent of the observed behavior in slices—regardless of the temporal aspect (Fig 6G).

Furthermore, to further assess the differences and similarities between beta cell networks from slices and isolated islets and how they relate to different types of oscillatory activity, we present in S3 Fig an analysis of an isolated islet where the fast component of oscillations was relatively well present, which is frequency-wise highly comparable to that in tissue slices. This enabled the separate consideration of individual oscillatory components, and similarly to tissue slices, it was found in this case as well that there is a significant similarity between the structural network and the functional network obtained from the fast component, while the similarity between the slow and structural is considerably lower. It is also worth mentioning that in isolated islets, there is much greater similarity between networks derived from unprocessed signals and the slow component, whereas in tissue slices, there is greater similarity between networks based on unprocessed signals and the fast component. The reason for this is that in tissue slices, fast oscillations are the more dominant type of signal, while in isolated islets, slow oscillatory activity prevails.

## Discussion

Functional connectivity analysis is a powerful tool applicable to studying the interactions between different components in a plethora of real-life systems. In recent years, it is becoming increasingly more popular to describe interactions between individual cells, particularly within the islets (for review see [56]). However, due to relatively demanding computational approaches, encompassing both data extraction and subsequent analyses of coordinated functioning, obtaining patterns of functional connectivity is not straightforward and can easily become ambiguous. In neuroscience, where the greatest progress in this field has been made, it has become evident that objectively assessing connectivity patterns is challenged by various objective reasons tied to experimental variations and computational methodologies, such as thresholding techniques [82–84], techniques used for data pooling [85,86], number of sensors used to record brain activity [87,88], and the selection of frequency intervals [89,90]. Most importantly, similar issues are witnessed in the network-based analysis of spatiotemporal cellular dynamics in islets. More specifically, different research groups employ diverse experimental techniques and preparations leading to discrepancies in types of oscillatory signals and the multicellular activity is recorded at varying spatial and temporal resolutions. There are also variations in how recordings are preprocessed before network analysis, as well as in the techniques used for the analysis itself. These, along with some terminological discrepancies in the scientific literature, are the primary reasons why we chose to investigate how various factors influence network analyses and their interpretation.

First, we evaluated the role of metrics that are used for the evaluation of synchronized activity between the measured cellular dynamics. We compared three different methods, namely one that is based directly on the recorded [Ca^2+^]_i_ activity (Pearson’s correlation), and two that are based on binarized time series (coactivity and mutual information). It turned out that irrespective of the method used to quantify synchronous behavior, similar networks are obtained, characterized by small-worldness, modularity, high degree of clustering, a heavy-tailed degree distribution which indicates the presence of hub cells, and a similar relation between the relative active time and the node degree (see Fig 1). Another crucial aspect in the process of extracting functional connectivity maps involves the thresholding of similarity matrices. As highlighted in Figs 2A and 3B, utilizing a fixed threshold can yield significant disparities among different islets, potentially introducing biases into the relations drawn from aggregated data. To mitigate this concern, using a variable threshold and a fixed average degree proves advantageous. With this approach we can firmly evaluate the effect of pharmacological interventions or extract the relations between network and classical physiological parameters when data is pooled from multiple islets, as a variable threshold can mask the inter-islet heterogeneity (see Figs 2C and 3). Specifically in multi-phase experiments, where consecutive intervals have to be analyzed [71,91], application of a variable threshold has proven beneficial, as it overcomes inter-islet variability. For example, by establishing the variable threshold based on the first interval, thereby maintaining a fixed average node degree, one can consistently apply the same threshold to construct networks during the second interval. This normalization procedure facilitates an objective assessment of alterations in islet network structure, despite inherent differences in networks from different islets (Fig 3). It is important to note, however, that this method has a limitation: its fixed average number of connections prevents it from capturing the variations in overall synchronicity that are depicted by the network density. For instance, it is known that an increase in glucose concentration leads to increased and more global spatiotemporal activity, resulting in denser functional networks [27,72]. If a fixed average degree is then employed, these differences become obscured, and in conditions of low stimulation, numerous connections emerge that lack statistical significance. This occurs because, with a low threshold, these connections predominantly signify random associations rather than synchronized activity (S1 Fig). In this study we have also introduced a third option encompassing the construction of functional networks through a multilayered MST. A notable advantage of this method lies in its absence of explicit thresholding, with the singular free parameter being the number of layers, which in turn specify the average degree. Nonetheless, the drawback of the minimum spanning tree method is that it enforces at least one connection to each cell (or more in case of multilayered MST), so that even the cells which are completely desynchronized can have a comparable number of functional connections as an average cell. Therefore, while the method is attractive for its apparent objectivity, its appropriateness diminishes when the signals are rather heterogeneous and if there are subpopulations of cells whose dynamics are weakly or not at all correlated with the rest of the cells (such as those of alpha cells, see S4 Fig). To sum up at this point, the choice of the best method to construct networks is not always straightforward and may depend on the context, i.e., both the experimental protocol and the parameters we want to objectively describe through network analysis. In doing so, we must, of course, be aware of the strengths and weaknesses of different approaches.

In previous studies, variations in glucose-induced [Ca^2+^]_i_ activity among different mouse strains and substrains have been reported. Compared to outbred NMRI mice, cells from the inbred C57BL/6J and C57BL/6N substrains show a rightward shift in activations and earlier deactivations. In addition, during the plateau phase, the encoding mechanisms to enhance calcium activity in response to glucose differ quantitatively in all three groups [33]. Secretagogues other than glucose also cause [Ca^2+^]_i_ oscillations to vary greatly [76]. Generally, however, there are similarities between C57BL/6J, C57BL/6N, and NMRI mice in the sense that all three groups showed glucose-dependent activation and deactivation responses, as well as a 3% increase in relative active time per millimole of glucose [33]. Notably, up until now, differences between strains of mice at the level of multicellular activity have not been studied. In this study, we addressed these questions using network analyses and found that the functional networks of islets in different mice are structurally very similar. Apparently, the mechanisms that coordinate fast oscillatory activity across the islets from NMRI or C57BL/6N mice, i.e., gap-junction mediated depolarization and [Ca^2+^]_i_ waves, are the same and do not differ between mouse strains.

In response to glucose and many other secretagogues, electrical activity, intracellular calcium, and insulin secretion oscillate in synchrony at two different time scales [92,93]. The first are the so-called metabolic or slow oscillations with a frequency of around 0.1–0.2 min^-1^, and the second the so-called electrical or fast oscillations with a frequency of around 1–5 min^-1^ [53,94]. Noteworthy, fast oscillations show variations and have the highest frequency rates around the peaks of the slow component and the lowest around the nadirs [77,95,96]. Additionally, the relative active time or duty cycle of the fast component characteristically increases with increasing stimulation, whereas the frequency of slow oscillations remain unaltered [27,33,96–98]. According to the recent metronome model of beta cell function, slow oscillations set the pace for insulin pulses, whereas the fast oscillations fine-tune their amplitude [94]. Both slow and fast oscillations are phase-locked between different beta cells within a given islet by means of intercellular waves [14,34,35,55,62,68]. In accordance with this, the average correlation between calcium traces of different cells from the same islet decreases with intercellular distance for both the slow and the fast component, implying that intercellular coupling mediates the synchronicity of both types of oscillations [78,96].

If one constructs and compares functional connectivity maps for the raw signal and both dynamic components separately (Fig 5), the distributions of node degrees do not differ significantly. However, the networks of fast oscillatory activity are more locally clustered and segregated, more modular, and have lower average edge lengths and global efficiency, while the slow oscillations are principally more global, resulting in numerous long-range connections and consequently a more cohesive structure that shows a lower modularity and higher global efficiency. Importantly, for the raw signal, it seems that except for the node degree, most of the network measures are more strongly determined by the slow component [56]. A logical consequence of the abovementioned differences in functional network structure is the finding that there is a relatively weak correlation between the fast and slow network layer [96], implying that different synchronization principles are at work [70,78], and one should not directly compare results of studies relying on fast oscillations with the ones relying on slow oscillations. Importantly, even with the same experimental model, e.g., isolated mouse islets, and set of analytical tools applied to extracting and analyzing [Ca^2+^]_i_ oscillations, islets with preponderance of fast, mixed or slow oscillations might coexist [99–101], and in this case, data should not be simply pooled, since this may obscure relevant biological differences, but analyzed for the two temporal components and for oscillatory phenotypes separately. Extrapolating this reasoning further, the caveats we pointed out in this paragraph should also be kept in mind when comparing experimental traces from different animal models, even when using the same experimental approach and the same set of analytical tools. For instance, the presence and relative importance of fast and slow oscillations may vary between beta cells from zebrafish [55,102], mice [100,103], rats [104,105], sand rats [106,107], pigs [108,109], and humans [61,110], to name only a few. To facilitate interspecies comparison, future studies shall clearly specify the type of oscillations they are addressing. Finally, at present, it is difficult to experimentally compare the relationship between the structural networks of beta cells and their functional counterparts, but modelling studies suggest that the intricate structure of functional beta cell networks based on fast and slow oscillations may be at least partly explained by heterogeneity in beta cell activity and heterogenous intercellular coupling [68,70,78].

Different groups that employ network measures in their analyses typically use different experimental approaches to obtain [Ca^2+^]_i_ traces. While most groups use cultured isolated islets in combination with CCD camera-based or confocal imaging, some use the acute tissue slices in combination with confocal imaging. To be able to compare findings from different groups or combine them into a coherent bigger picture of islet network properties, these differences also need to be addressed as they are an important possible systematic confounding variable. Essentially, the methodology and experimental setup would not seem to be key parameters if they did not entail differences in the nature of the oscillatory signals. In tissue slices fast or mixed oscillations are more predominant (see Figs 4A or 5A), whereas in isolated islets the slow oscillations are predominant (see Fig 6A). Here, we explicitly demonstrated that the distinct nature of oscillations leads to different functional beta cell networks. While some network properties in fast-derived and slow-derived networks are similar, such as heterogeneity and small-worldness, they fundamentally differ from each other, and the significance of certain subpopulations in one network is therefore not equivalent to that in the other network. Moreover, even if oscillations qualify as fast, in isolated islets, they are typically longer than 10 seconds at concentrations > 10 mM glucose [34,92,99], whereas in slices, they tend to be shorter than 10 seconds [16,27,33,111]. The exact mechanism behind these differences remains to be explained, but in addition to possible differences in ionic composition and the presence of additional secretagogues in the extracellular fluid that can affect the patterns of oscillations [92,112,113], the mechanical and enzymatic stress during preparation of isolated islets [114,115], as well as culture conditions and duration [99,116] have been put forward as possible sources of these differences. More specifically, alpha cells have been suggested as a potential source of local proglucagon peptides [117]. They are primarily situated in the mantle of pancreatic islets in mice, and this outer region is particularly susceptible to damage during the islet isolation process, potentially resulting in the loss of alpha cells during islet preparation. Given that both glucagon and GLP-1 have been shown to elevate the frequency of oscillations in beta cells, the diminished intra-islet alpha cell signalling could be a contributing factor to the observed decrease in beta cell oscillatory frequency in isolated islets [71,91,118,119]. Further, there may be a run-down of certain ion channels and changes in the expression [120, 121] with time, which obviously impact the identity and physiology of beta cells in the cultured isolated islets more than in the immediately used islets in slices [122,123]. This theory is at least partly supported by the finding that oscillations in mouse islets cultured for less than one day closely resemble oscillations in non-cultured islets [103,124] and in islets studied *in vivo* [125,126] or rapidly after the death of the animal [92], as well as the oscillations in tissue slices [27]. Until the influence of the above factors is fully understood, we can provide at least two practical suggestions. First, studies on isolated islets and tissue slices should always exactly state what the composition of the extracellular fluid was, and which type of oscillations were used for the network analyses, as well as provide details about the basic characteristics of these oscillations, i.e., their frequency and duration. Second, freshly microdissected islets or islets cultured for shorter time periods may yield results that are more closely comparable with results from tissue slices. Finally, the above advice also applies for studies utilizing yet other experimental preparations, such as *in vivo* imaging of isolated and transplanted mouse islets in the anterior chamber of the eye [127] and islets from other species, as mentioned in the preceding paragraph. In the present study, we used a range of different stimulatory concentrations. They are not intended to illustrate possible glucose-dependencies of different physiological and network metrics as these are covered elsewhere [13,27,33], but to demonstrate that the analytical tools work robustly across a range of frequently used stimulatory conditions. Given that the slow oscillations are rather glucose-insensitive in terms of their frequency in both slices [96] and islets [98] and that fast oscillations have comparable dose-response relationships in slices [27,33] and isolated islets [128–130], we believe that the different concentrations we used did not introduce any critical bias and that most of our findings are applicable to concentrations beyond the range used here.

In conclusion, we would like to stress that the scope of network analyses has, in recent years, been extended to investigate intercellular interactions and functional connectivity patterns in different types of tissues. These encompass various kinds of neural assemblies [131], pituitary endocrine cells [132,133], astrocytes [134], yeast cells [135], distinct epithelial cell types [136,137], acinar cells [138], and hepatocytes [139]. As such, the insights we present herein hold relevance for comprehending the intricacies of collective cellular activity across diverse contexts, where the assessment of multicellular dynamics can be achieved through suitable imaging techniques. Moreover, in tandem with advancements in imaging methods, which are expected to soon enable the simultaneous high-resolution assessment of multiple variables defining multicellular activity, potentially even in three dimensions, it is imperative to stay attuned to progress on the computational front. Over recent years, a plethora of sophisticated methods has emerged for evaluating dynamic interactions within complex systems, such as multilayer networks [140,141], detection of higher-order interactions [142,143], information-theoretic metrics describing causal relationships [144,145], and deep learning-based methods [146,147]. These approaches hold substantial potential for further and more profound research, extending even into the realm of multicellular systems, as already demonstrated by some recent studies [13,56,148–150]. We strongly believe that future progress in this field will rely on such interdisciplinary endeavors that combine cutting-edge experiments with innovative computational procedures. Along these lines, we anticipate a deeper understanding of how heterogeneous populations of interacting cells, placed within a dynamic and noisy environment, operate to ensure proper functionality, and how the regulatory mechanisms are altered in disease.

## Materials and methods

### Ethics statement

We conducted the study in strict accordance with all national and European recommendations on care and handling experimental animals, and all efforts were made to minimize the suffering of animals. Mice were used under protocols approved by the University of Colorado Institutional Animal Care and Use Committee (IACUC Protocol number: 00024) and The Administration of the Republic of Slovenia for Food Safety, Veterinary and Plant Protection (permit numbers: U34401-35/2018-2).

### Animals and [Ca2+]i imaging in tissue slices

#### Slice preparation

C57Bl6J and NMRI male and female mice were held in a temperature-controlled environment with a 12 h light/dark cycle and given continuous access to food and water. Preparation of mouse-derived acute pancreas tissue slices was executed as described previously in full [122]. In brief, after sacrifice with CO_2_ and cervical dislocation, the abdominal cavity is accessed via laparotomy and the papilla Vateri is clamped. 1.9% Low melting agarose dissolved in ECS containing (in mM) 125 NaCl, 26 NaHCO3, 6 glucose, 6 lactic acid, 3 myo-inositol, 2.5 KCl, 2 Na-pyruvate, 2 CaCl2, 1.25 NaH2PO4, 1 MgCl2, 0.5 ascorbic acid is heated to 40°C and injected through the bile duct. The pancreas is cooled with ice-cold ECS, extracted, and cut into tissue blocks, which are embedded in low melting point agarose and cut with a vibratome (VT 1000 S, Leica) to yield 140 μm slices. The slices are kept in HEPES-buffered saline (HBS) consisting of (in mM) 150 NaCl, 10 HEPES, 6 glucose, 5 KCl, 2 CaCl2, 1 MgCl2; titrated to pH = 7.4 with 1 M NaOH at room temperature and stained with a HBS staining solution containing 7 μM Calbryte 520 AM (AAT Bioquest), 0.03% Pluronic F-127 (w/v), and 0.12% dimethyl sulfoxide (v/v) for 50 min at room temperature. All chemicals were obtained from Sigma-Aldrich (St. Louis, Missouri, USA) unless stated otherwise. Individual tissue slices were placed into the recording chamber and used for one stimulation protocol. The recording chamber was continuously perifused with carbogenated ECS containing 6 mM glucose heated to 37°C at basal conditions. At 20–40 minutes, the perifusion was manually changed to stimulatory (8–12) mM glucose before it was returned to the basal glucose concentration.

#### Imaging

Beta cell calcium dynamics were imaged using an upright confocal microscope system Leica TCS SP5 AOBS Tandem II with a 20X HCX APO L water immersion objective, NA 1.0, and an inverted confocal system Leica TCS SP5 DMI6000 CS with a 20X HC PL APO water/oil immersion objective, NA 0.7 (all from Leica Microsystems, Germany). A 488 nm argon laser was used to excite the fluorescent dye, and a Leica HyD hybrid detector operating in the 500–700 nm range was used to detect the fluorescence that was released (all from Leica Microsystems, Germany), as previously described [27,122]. The resolution used for time series acquisition was 512 X 512 pixels with a frequency of 2–10 Hz.

### [Ca^2+^]_i_ imaging in isolated islets

#### Islet isolation and culture

Islets were isolated from mice under ketamine/xylazine anaesthesia (80 and 16 mg/kg) by collagenase delivery into the pancreas via injection into the bile duct. The collagenase-inflated pancreas was surgically removed and digested. Islets were handpicked and planted into the glass-bottom dishes (MatTek) using CellTak cell tissue adhesive (Sigma-Aldrich). Islets were cultured in RPMI medium (Corning, Tewksbury, MA) containing 10% fetal bovine serum, 100 U/mL penicillin, and 100 mg/mL streptomycin. Islets were incubated at 37C, 5% CO2 for 24–72 h before imaging.

#### Imaging

An hour prior to imaging nutrition media from the isolated islets was replaced by an imaging solution (125 mM NaCl, 5.7 mM KCl, 2.5 mM CaCl2, 1.2 mM MgCl2, 10 mM HEPES, and 0.1% BSA, pH 7.4) containing 2 mM glucose and fluo4 AM [Ca^2+^]_i_ sensitive dye (4 mM). After one hour the solution was replaced by dye-free imaging solution. During imaging the glucose level was raised from 2 mM to 11 mM. Islets were imaged using either a LSM780 system (Carl Zeiss, Oberkochen, Germany) with a 40x 1.2 NA objective or with an LSM800 system (Carl Zeiss) with 20x 0.8 NA PlanApochromat objective or a 40x 1.2 NA objective, with samples held at 37°C. The resolution was 512x512 pixels and time series were recorded with frequencies 1–2 Hz.

### Pre-processing of recorded [Ca^2+^]_i_ time series

Fluorescence signals of Calbryte 520 AM or Fluo-4 representing time series for manually selected regions of interest (ROIs), i.e., individual beta cells, were exported along with their corresponding coordinates using a custom software called ImageFiltering (copyright Denis Špelič) or ImageJ [151]. As both dyes can detect both fast- and slow-component in beta [61,152], data obtained by either dye was pre-processed equally. Time series that exhibited large artifacts, low signal-to-noise ratio, or dynamics inconsistent with beta cells were excluded after visual inspection. The recordings from tissue slices underwent band-pass filtering using a zero-lag filter to extract either the fast-activity component (with typical cut-off frequencies of 0.05 and 2.0 Hz) or the slow-activity component (with cut-off frequencies of 0.001 and 0.07 Hz). Similarly, the recordings from isolated islets underwent band-pass filtering to eliminate baseline drifts and capture the oscillatory component (with typical cut-off frequencies of 0.005 and 0.25 Hz). Fast-component signals from slices and oscillatory signals from isolated islets were further smoothed using an adjacency averaging procedure and then binarized by setting values to 1 (active state) for periods of increased [Ca^2+^]_i_ signals or 0 (inactivity) for periods of low-amplitude signals. All subsequent analyses were performed either on the raw, filtered (fast or slow oscillatory component), or binarized cellular signals. The binarized signals were also used to calculate the relative active time. This metric represents the ratio of the time a given cell is in an active state, indicating thereby the overall cellular activity.

### Evaluating synchronicity between [Ca^2+^]_i_ traces

We use three different methods to quantify the similarity between recorded [Ca^2+^]_i_ traces: the Pearson correlation, the coactivity, and the mutual information. The latter two are computed on the basis of binarized traces, whereas the Pearson correlation is computed on the basis of filtered traces. The Pearson correlation coefficient (*PC*_*i*,*j*_) between the *i*-th and *j*-th cell quantifies the linear relationship between the corresponding [Ca^2+^]_i_ traces and is computed as:

$$PC_{i,j}=\frac{\sum \left({\bar{x}}_{i}-x_{i}(t))({\bar{x}}_{j}-x_{j}(t)\right)}{\sigma_{x_{i}}\sigma_{x_{j}}},$$ (1)

where *x*_*i*_ and *x*_*j*_ are recorded time series for cells *i* and *j*, respectively, and σ represents their corresponding standard deviations. Eq (1) generates a value between -1 and 1, where -1 indicates completely anticorrelated time series and 1 implies identical time series. Although this is a popular similarity measure, it has some limitations. It cannot capture nonlinear relationships and can result in very high values in coupled oscillator systems like beta cell networks, which can limit its usefulness. Another popular method for measuring time series similarity is coactivity [132,153]. This method relies on binarized time series and measures the degree of simultaneous activity between cell pairs. It corresponds to the dot product of two normalized binary time series vectors, where each data point is one dimension of the vector. The coactivity coefficient between cell pairs *i* and *j* is calculated as:

$$CA_{i,j}=\frac{\sum \left(x_{b,i}x_{b,j}\right)}{\sqrt{\sum x_{b,i}}\sqrt{\sum x_{b,j}}},$$ (2)

where *x*_*b*,*i*_ and *x*_*b*,*j*_ are the binarized activity time series of cells *i* and *j*, respectively. This measure gives a value of 0 if there is no overlap in activity and 1 if the cells are completely coactive. The method quantifies the overlap of [Ca^2+^]_i_ oscillations in a very straightforward manner but does not capture nonlinear relationships between cells.

Mutual information (*MI*) is another commonly used method to measure the similarity of time series [154]. It is a statistical measure that quantifies the amount of information shared between two random variables and the degree of dependence between them. In essence, it measures how knowledge about one variable can help predict the other. Mutual information is calculated based on the Shannon entropy of both time series (*H*(*x*_*b*,*i*_) and *H*(*x*_*b*,*j*_)) and their joint entropy (H(*x*_*b*,*i*_,*x*_*b*,*j*_)). For binarized time series of the cell pair *i* and *j*, *MI* is expressed as follows [155]:

$$MI(x_{b,i},x_{b,j})=H(x_{b,i})+H(x_{b,j})-H(x_{b,i},x_{b,j}).$$ (3)

The value obtained by Eq (3) ranges from 0 (independent time series) to 1 (completely co-dependent time series). The Shannon entropy (*H*) of binarized time series *i* (*x*_*b*,*i*_) is defined as [155]:

$$H(x_{b,i})=-\sum_{s\in xb_{i}}p(s_{i})\log_{2}\left(s_{i}\right)$$ (4)

where *p*(*s*_*i*_) is the probability distribution that *x*_*b*,*i*_ takes one of its possible values (*s*_*i*_∈[0,1]). Values for *p*(*s*_*i*_) can be easily calculated based on the frequency count (*F*(*s*_*i*_)) of individual values *s*_*i*_ and the length (*L*) of the time series (*p*(*s*_*i*_) = *F*(*s*_*i*_)/*L*). The joint entropy of two binarized time series *x*_*b*,*i*_ and *x*_*b*,*j*_ is then calculated as [155]:

$$H(x_{b,i},x_{b,j})=-\sum_{s_{i}\in xb_{i}}\sum_{s_{j}\in xb_{j}}p(s_{i},s_{j})\log_{2}p(s_{i},s_{j}).$$ (5)

In the case of two binarized time series, there are four possible value combinations for (*s*_*i*_,*s*_*j*_). To ensure comparability between values of different cell pairs, the normalized mutual information (${\tilde{MI}}_{i,j}$) is calculated as:

$${\tilde{MI}}_{i,j}=\frac{MI(x_{b,i},x_{b,j})}{\sqrt{H(x_{b,i})H(x_{b,j})}}.$$ (6)

By using Eqs (1), (2), and (6), we can construct similarity matrices of size (*N*, *N*), whereby *N* stands for the number of cells, that encode the correlation, coactivity, and normalized mutual information between all cell pairs in individual recordings, respectively. Notably, *MI* captures also non-linear relationships between the discretized time series.

### Network construction and analysis

The functional networks are extracted from similarity matrices, whereby different approaches can be used to this purpose. The most elemental way is to set up a predetermined threshold, so that a connection between a cell pair *i* and *j* is established only if their similarity coefficient (*SC*_*i*,*j*_) exceeds the pre-set similarity threshold (*SC*_th_). Specifically, cells *i* and *j* are connected if:

$$SC_{i,j}>SC_{th},$$ (7)

where *SC*_*i*,*j*_ is based on one of the aforementioned similarity measures.

Alternatively, a variable similarity threshold technique can be used instead of a fixed threshold, which can create a network with a pre-set target average node degree, so the threshold is varied until a network with the target average node degree is designed. In our analyses the variable threshold was determined so that the resulting network had an average degree 8. This value was used to mimic the connectivity of realistic beta cell network architectures [156] and to obtain adequately dens networks suitable for analyses. However, it should be noted that previous studies have demonstrated that, within reasonable limits, the conclusions drawn from network analyses are not significantly influenced by the somewhat arbitrary choice of the average degree [13,96].

The third option is to utilize the multilayer minimum spanning tree (multilayer MST). In graph theory, a minimum spanning tree is defined as a graph that connects all the nodes with the minimum possible total edge weight, without forming any cycles. To construct the MST, the similarity coefficients of cell pairs (*SC*_*i*,*j*_) must be recomputed to an abstract distance measure (*D*_*i*,*j*_) using the following eq:

$$D_{i,j}=\sqrt{2(1-SC_{i,j})}.$$ (8)

Based on the computed abstract distances, an MST can be constructed with so-called greedy algorithms such as Kruskal’s [157] or Prim’s [158] algorithm. These algorithms create graphs with *N*-1 edges (*N*–number of nodes) which contain the lowest possible sum of edge weights (Σ*D*_*i*,*j*_) without creating any cycles. We expand this idea for the generation of a multilayer MST, where a single MST is computed sequentially for the same network, but already existing edges (i.e., cell pairs) are excluded from the calculation of the next MST layer. In our analyses we calculated four layers of MST’s, which yielded an average node degree of 8 (the average degree of the original MST is 2, and each of the three subsequent layers contributes an additional 2 degrees).

For each extracted network, we calculated several basic network parameters, such as average network node degree (*k*_avg_) and degree distribution, average clustering coefficient (*C*_avg_) and clustering coefficient distribution, modularity (*Q*), global efficiency (*E*), relative largest component (*S*_max_), and edge length distribution, and small-world coefficient (*SW*). See Ref. [159] for technical details and Ref. [56] for a physiological meaning of these specific network parameters.

### Quantifying inter-network similarity

To quantify the similarity between networks constructed with different approaches, we computed the network similarity index (*NSI*) based on the sets of edges (*A*) for each individual network (*α*). For network pairs *α* and *α*′ NSI was calculated using the Jaccard similarity coefficient as follows:

$${NSI}_{\alpha ,\alpha \prime }=\frac{\left|A_{\alpha }\cap A_{\alpha \prime }\right|}{\left|A_{\alpha }\cup A_{\alpha \prime }\right|}.$$ (9)

In other words, inter-network similarity is defined as the ratio between the cardinality, i.e., the total number of edges of the intersection of edges in networks *α* and *α*′, and the cardinality of the corresponding union. The resulting value of *NSI* ranges from 0 to 1, where 0 indicates no common edges between the networks and 1 indicates identical networks. This method was used to assess the similarity between functional networks derived from various oscillatory components and constructed with the above-described construction techniques. We additionally quantified the similarity of these networks with the postulated structural networks of islet cells, which we constructed as geometric networks by appropriate intercellular distance thresholding.

Methods for the time series processing, analyses of cellular signals, and network analyses were designed with Python programming language version 3.11.1, using the following packages: Numpy (https://numpy.org/), Matplotlib (https://matplotlib.org/), and NetworkX (https://networkx.org/). All code is available on the GitHub repository: https://github.com/MarkoSterk/beta_cell_analysis_suite

## Supporting information

S1 Fig Collective beta cell activity under the protocol of a glucose ramp. A) Ca^2+^ traces of all responding beta cells in the slice (upper panel) and the corresponding raster plot of binarized fast Ca^2+^ oscillations. The glucose concentration was ramped from 6 mM to 12 mM, as indicated at the top. B) Functional beta cell networks extracted in different glucose concentrations and with different thresholding techniques. The fixed threshold approach (*R*_th_ = 0.8) leads to very different network structures under different stimulation levels. Under lower glucose, when the degree of correlated beta cell dynamics is low, the networks are sparse and segregated. With increasing stimulation, the networks become progressively more integrated and dense (i.e., average node degree *k*_avg_ is increasing), highlighting the heightened intercellular coordination. Conversely, the fixed avg. degree and multilayer MST approaches fail to capture this behavior, as they enforce a fixed number of connections, irrespective of the level of coordinated intercellular activity. Furthermore, utilizing a fixed average degree under conditions of low multicellular activity results in exceedingly low thresholds (*R*_th_ < 0.5), thereby promoting the establishment of functional connections by chance, which introduces unpredictability into the network analysis. Consequently, techniques that enforce a fixed number of connections are unsuitable for experiments where the level of activity changes significantly.
(TIF)

S2 Fig Exploring the Impact of Oscillatory Components and Calcium Signal Processing on Functional Network Structure. The figure presents four types of networks derived from analysis of the five different islets examined in Fig 5: i) A structural network modelled as a geometric network, wherein nearby cells are deemed connected. ii) A functional network derived from unprocessed signals. iii) A functional network extracted from the fast oscillatory component. iv) A functional network constructed based on the slow oscillatory component. All four networks were designed with a fixed average degree *k*_avg_ = 8. Remarkably, across all five islets, the functional network based on the fast oscillatory component exhibits the fewest long-range connections and shows the highest similarity to the hypothesized structural network. In contrast, networks derived from unprocessed or slow-component signals display a greater proportion of long-range connections, exhibit similar characteristics to each other, and diverge significantly from the structural network.
(TIF)

S3 Fig Investigating the influence of oscillatory component on functional network structure in an isolated islet. A) The average unprocessed (black) and extracted slow-component Ca^2+^ signal (blue) from a Gcamp mouse islet are depicted. The inset shows the corresponding derived fast-component signal (red). B) Different types of beta cell networks: structural (modelled as a geometric network) and three functional networks derived from the unprocessed, slow-component, and fast-component Ca^2+^ dynamics. Hub cells are highlighted in red. C) Inter-network similarity matrix quantifying the degree of overlap between the four networks. Evidently, the networks extracted from the unprocessed and slow-component traces are very similar, while the fast component network exhibits the highest degree of similarity with the structural network. In contrast, the similarity between the networks derived from unprocessed and slow-component signals and the structural network is notably lower, mirroring observations in tissue slices (see Figs 6 and S2).
(TIF)

S4 Fig Comparative analysis of functional intercellular network design methods. A) Three representative beta cell signals (red line) and three alpha cell signals (blue line) subjected to the indicated stimulation protocol: 9 mM -> 10 mM -> 11 mM -> 11 mM glucose + μM epinephrine. This protocol was used to functionally discriminate alpha and beta cells, as the addition of 1 μM epinephrine activates alpha cells and inhibits beta cells. B) Functional networks were extracted using two methods: the fixed average degree method (left) and the four-layered multilayer minimum spanning tree (MST) method (right). The multilayer MST method enforced connections to all cells, including those with asynchronous dynamics, such as alpha cells. Consequently, alpha cells were integrated into the functional network despite their lack of correlation with the rest of the syncytium. This highlights the unsuitability of the MST method for network analyses involving elements with diverse dynamics. Alpha cells are indicated with blue circles and beta cells with red circles.
(TIF)
