# Supplementary figures and images for: Network representation of multicellular activity in pancreatic islets: Technical considerations for functional connectivity analysis
Source: PLoS Comput Biol. 2024 May 13;20(5):e1012130. doi: 10.1371/journal.pcbi.1012130 (PMC11115366; doi:10.1371/journal.pcbi.1012130)

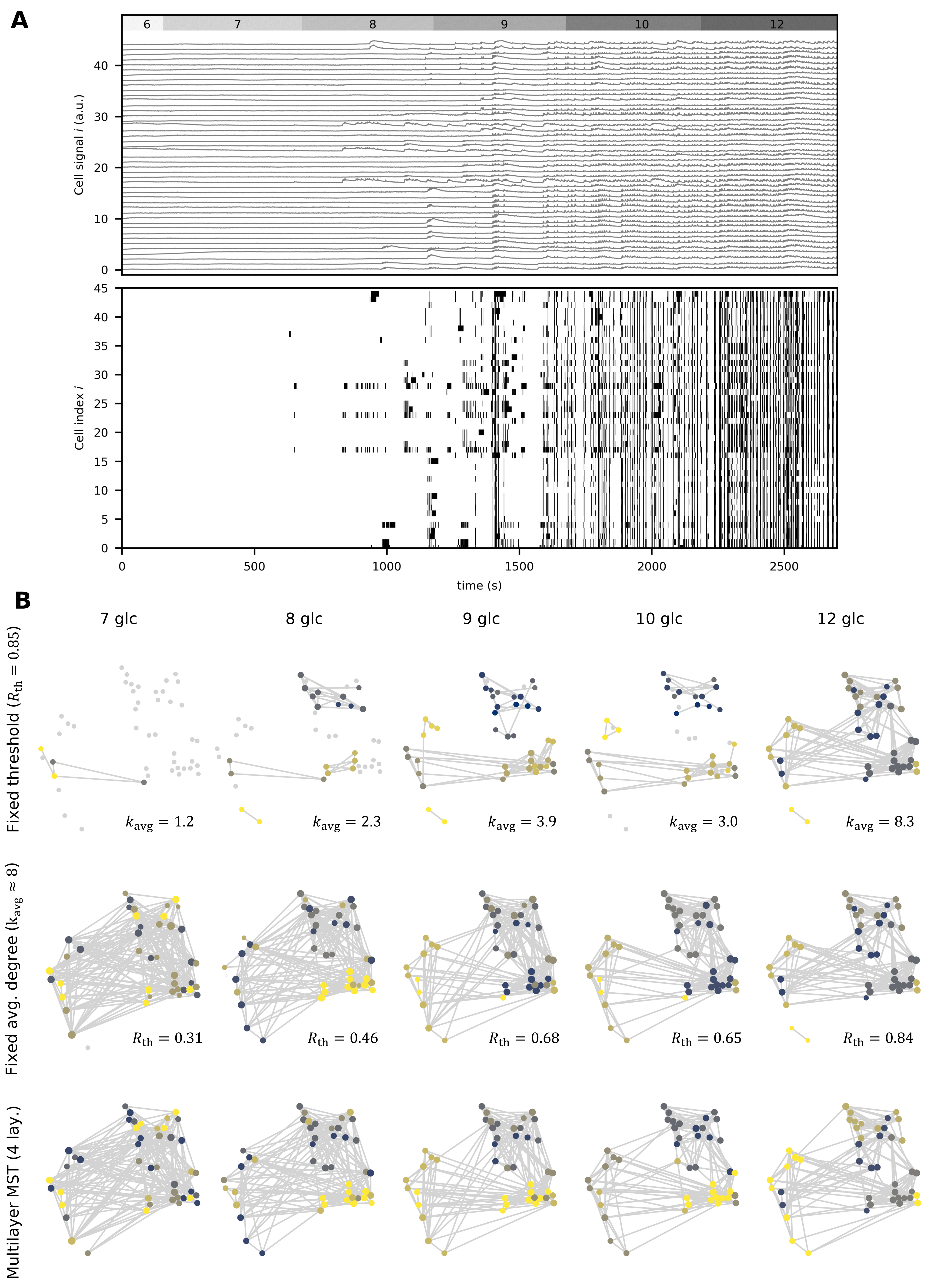

Supplement: S1 Fig — A) Ca2+ traces of all responding beta cells in the slice (upper panel) and the corresponding raster plot of binarized fast Ca2+ oscillations. The glucose concentration was ramped from 6 mM to 12 mM, as indicated at the top. B) Functional beta cell networks extracted in different glucose concentrations and with different thresholding techniques. The fixed threshold approach (Rth = 0.8) leads to very different network structures under different stimulation levels. Under lower glucose, when the degree of correlated beta cell dynamics is low, the networks are sparse and segregated. With increasing stimulation, the networks become progressively more integrated and dense (i.e., average node degree kavg is increasing), highlighting the heightened intercellular coordination. Conversely, the fixed avg. degree and multilayer MST approaches fail to capture this behavior, as they enforce a fixed number of connections, irrespective of the level of coordinated intercellular activity. Furthermore, utilizing a fixed average degree under conditions of low multicellular activity results in exceedingly low thresholds (Rth < 0.5), thereby promoting the establishment of functional connections by chance, which introduces unpredictability into the network analysis. Consequently, techniques that enforce a fixed number of connections are unsuitable for experiments where the level of activity changes significantly. (TIF) [file pcbi.1012130.s001.tif]

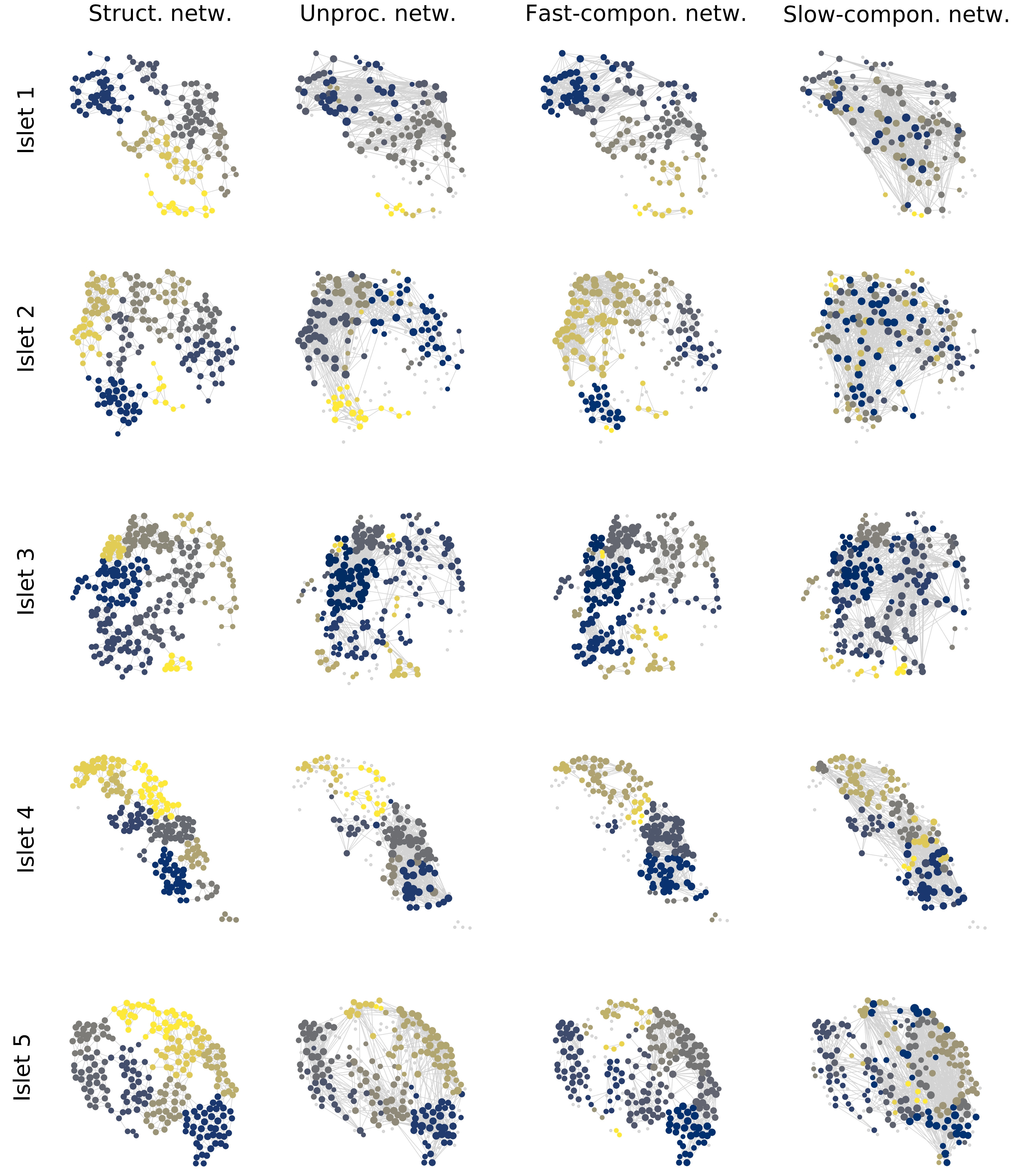

Supplement: S2 Fig — The figure presents four types of networks derived from analysis of the five different islets examined in Fig 5: i) A structural network modelled as a geometric network, wherein nearby cells are deemed connected. ii) A functional network derived from unprocessed signals. iii) A functional network extracted from the fast oscillatory component. iv) A functional network constructed based on the slow oscillatory component. All four networks were designed with a fixed average degree kavg = 8. Remarkably, across all five islets, the functional network based on the fast oscillatory component exhibits the fewest long-range connections and shows the highest similarity to the hypothesized structural network. In contrast, networks derived from unprocessed or slow-component signals display a greater proportion of long-range connections, exhibit similar characteristics to each other, and diverge significantly from the structural network. (TIF) [file pcbi.1012130.s002.tif]

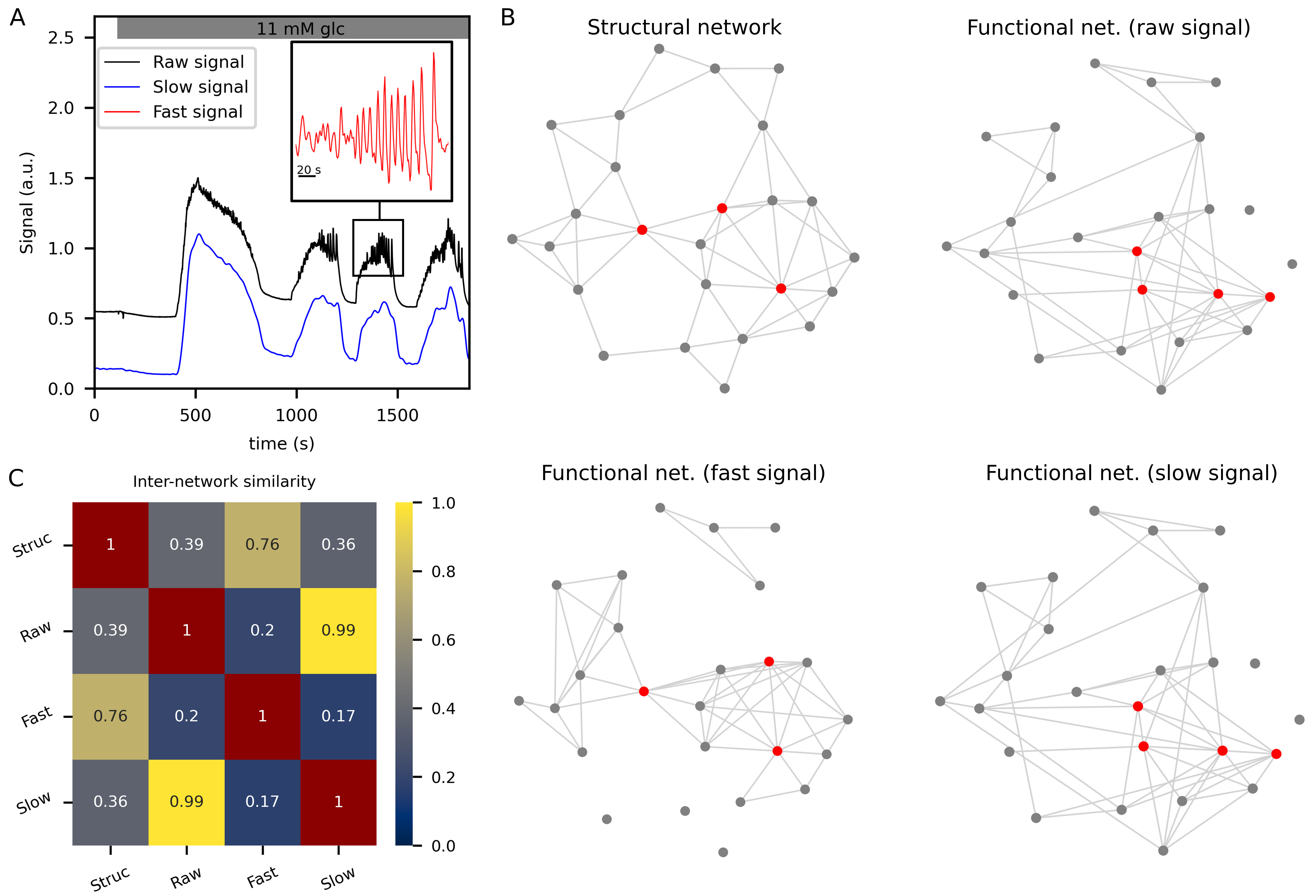

Supplement: S3 Fig — A) The average unprocessed (black) and extracted slow-component Ca2+ signal (blue) from a Gcamp mouse islet are depicted. The inset shows the corresponding derived fast-component signal (red). B) Different types of beta cell networks: structural (modelled as a geometric network) and three functional networks derived from the unprocessed, slow-component, and fast-component Ca2+ dynamics. Hub cells are highlighted in red. C) Inter-network similarity matrix quantifying the degree of overlap between the four networks. Evidently, the networks extracted from the unprocessed and slow-component traces are very similar, while the fast component network exhibits the highest degree of similarity with the structural network. In contrast, the similarity between the networks derived from unprocessed and slow-component signals and the structural network is notably lower, mirroring observations in tissue slices (see Figs 6 and S2). (TIF) [file pcbi.1012130.s003.tif]

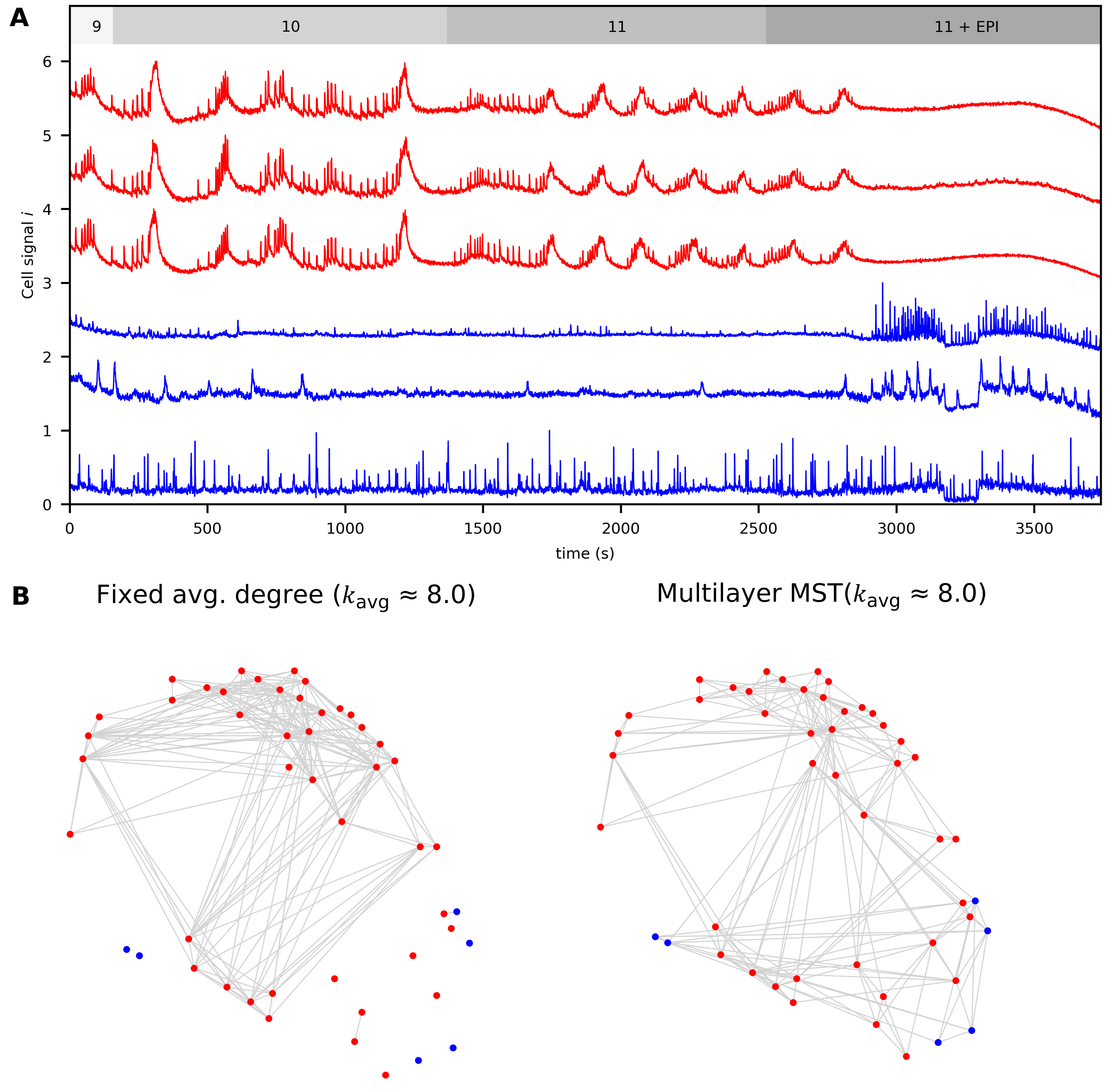

Supplement: S4 Fig — A) Three representative beta cell signals (red line) and three alpha cell signals (blue line) subjected to the indicated stimulation protocol: 9 mM -> 10 mM -> 11 mM -> 11 mM glucose + μM epinephrine. This protocol was used to functionally discriminate alpha and beta cells, as the addition of 1 μM epinephrine activates alpha cells and inhibits beta cells. B) Functional networks were extracted using two methods: the fixed average degree method (left) and the four-layered multilayer minimum spanning tree (MST) method (right). The multilayer MST method enforced connections to all cells, including those with asynchronous dynamics, such as alpha cells. Consequently, alpha cells were integrated into the functional network despite their lack of correlation with the rest of the syncytium. This highlights the unsuitability of the MST method for network analyses involving elements with diverse dynamics. Alpha cells are indicated with blue circles and beta cells with red circles. (TIF) [file pcbi.1012130.s004.tif]
